# Supplementary material for: Culture of Mycobacterium smegmatis in Different Carbon Sources to Induce In Vitro Cholesterol Consumption Leads to Alterations in the Host Cells after Infection: A Macrophage Proteomics Analysis
Source: Pathogens. 2021 May 28;10(6):662. doi: 10.3390/pathogens10060662 (PMC8230116; doi:10.3390/pathogens10060662)
Supplement: Supplementary file 1 [file pathogens-10-00662-s001.zip › S1_7H9.pdf]

Supplementary Table S1. List of differentially regulated proteins from the group of macrophages infected with *M. smegmatis* grown in complete 7H9 medium supplemented with glycerol (7H9 + Glycerol).

Uniprot acess code	Protein	*Fold-change*	Biologic Process	Molecular Function
	Down-regulated			
Q9WUM4	Coronin-1C	-1,383	Endosome Fission/ Phagocytosis	Binding Rac-GTPase / Binding actin filaments
P68373	Tubulin alpha-1C	-1,079	Microtubule Cytoskeleton Organization / Mitotic Cell Cycle	GTP binding / GTPase activity
	Up-regulated			
Q64337	Sequestosome-1 (Sqstm1/p62)	1,139	Autophagy receptor	Ubiquitin-binding protein p62
Q8K1N2	Pleckstrin homology-like domain family B member 2 (LL5-beta)	1,296	Microtubule Cytoskeleton Organization	assembly of the postsynaptic apparatus
Q60974	Nuclear receptor corepressor 1 (NCoR)	1,035	Nuclear receptor co-repression	DNA binding
Q9CQW9	Interferon-induced transmembrane protein (IFITM3)	1,545	Response to Interferon and receptor-mediated endocytosis	Role in the stability and function of vacuolar ATPase
	**Unique protein**			
G3X8Q1	Calcineurin binding protein 1 (Cabin1)	5,571	Negative regulation of cell death	Protein phosphatase 2B inhibitor activity
