# Supplementary material for: Culture of Mycobacterium smegmatis in Different Carbon Sources to Induce In Vitro Cholesterol Consumption Leads to Alterations in the Host Cells after Infection: A Macrophage Proteomics Analysis
Source: Pathogens. 2021 May 28;10(6):662. doi: 10.3390/pathogens10060662 (PMC8230116; doi:10.3390/pathogens10060662)
Supplement: Supplementary file 1 [file pathogens-10-00662-s001.zip › S2_MM+Chol.pdf]

Supplementary Table S2. List of differentially regulated proteins from the group of macrophages infected with *Mycobacterium smegmatis* grown in minimal medium supplemented with cholesterol (MM + Cholesterol).

Uniprot acess code	Protein	*Fold-change*	Biologic Process	Molecular Function
	Down-regulated			
P50516	V-type Proton ATPase (V-ATPase)	-1,1301	Transmembrane proton transport / Phagosome acidification	ATP binding/ Proton-transporting ATPase activity
G3UX26	Voltage-dependent anion-selective channel protein 2 (VDAC2)	-1,06274	Inorganic Transmembrane Anion Transport	Nucleotide Binding / Porin Activity
P11499	Heat shock protein HSP 90-beta (Hsp90ab1)	-1,25473	Protein folding, stabilization and degradation	ATP binding/ Chaperone
Q9WTX5	S-phase kinase-associated protein 1 (Skp1a)	-1,71938	Protein ubiquitination	Beta-catenin binding
Q8VDM4	26S proteasome non-ATPase regulatory subunit 2 (Psmd2)	-1,0267	Regulation of protein catabolic process	Enzyme regulator activity
P63028	Translationally-controlled tumor protein (Tpt1)	-1,72798	Proliferation of Cell Population	Calcium ion binding/Apoptosis
P17742	Peptidyl-prolyl cis-trans isomerase A (Ppia)	-1,79436	Lipid droplet organization	Cyclosporin A binding/ Unfolded protein binding
A5D6P2	Partitioning defective 3 homolog (Pard3)	-2,22628	Bicellular tight junction assembly/ Cellular migration	Identical protein binding/ Protein phosphatase binding
Q91Z83	Myosin-7 (Myh7)	-1,21607	Processo Metabólico de ATP	Actin-dependent ATPase activity

P62962	Profilin-1 (Pfn1)	-1,63522	Actin cytoskeleton organization	Actin binding
O88342	WD repeat-containing protein 1 (Wdr1)	-1,34062	Actin cytoskeleton organization	Actin binding
F8WGL3	Cofilin-1 (Cfl1)	-2,03354	Actin filament depolymerization	Actin binding
E9Q3V6	Septin-2 (Sept2)	-1,53592	Cytoskeleton-Dependent Cytokinesis	GTP binding/ Atividade de GTP
D6RD00	RNA 3'-terminal phosphate cyclase (Rtca)	-1,28617	RNA processing	ATP binding/ RNA-3'-phosphate cyclase activity
G3UY38	Heterogeneous nuclear ribonucleoprotein L (Hnrnpl)	-1,3193	mRNA processing	RNA binding
Q99KP6	Pre-mRNA-processing factor 19 (Prpf19)	-1,27397	mRNA splicing and DNA repair	Ubiquitin protein ligase activity
A0A0R4J259	Heterogeneous nuclear ribonucleoprotein Q (Syncrip)	-1,35788	mRNA processing	RNA binding
O08663	Methionine aminopeptidase 2 (Metap2)	-1,15093	Peptidyl-methionine modification	Aminopeptidase activity
P08030	Adenine phosphoribosyltransferase (Aprt)	-1,67802	Adenine metabolic process	AMP binding/ Adenine binding
Q8BMJ2	Leucine--tRNA ligase, cytoplasmic (Lars)	-2,35294	Cellular response to amino acid starvation	Leucine-tRNA ligase activity
P40142	Transketolase (Tkt)	-1,35548	Glyceraldehyde-3-phosphate biosynthetic process	Transketolase activity
Q9DBJ1	Phosphoglycerate mutase 1 (Pgam1)	-2,1921	Glycolytic process	Bisphosphoglycerate mutase activity

Q9JII6	Aldo-keto reductase family 1 member A1 (Akr1a1)	-2,63925	Oxidation and Reduction Process	Oxidoreductase activity
Q571I9	Aldehyde dehydrogenase family 16 member A1 (Aldh16a1)	-1,07626	Oxidation and Reduction Process	Oxidoreductase activity
P45377	Aldose reductase-related protein 2 (Akr1b8)	-1,34839	Oxidation and Reduction Process	Alcohol dehydrogenase (NADP+) activity
Q7TT23	Uncharacterized protein	-1,0289		
	Up-regulated			
Q6P9L6	Kinesin-like protein KIF15 (Kif15)	1,468724	Microtubule-based movement	ATP binding/ ATPase activity
P20152	Vimentin (Vim)	1,380619	Intermediate filament organization	Structural constituent of cytoskeleton
O35639	Annexin A3 (Anxa3)	1,331567	Neutrophil degranulation/ Phagocytosis	Calcium ion binding
Q9D2G2	Dihydrolipoyllysine-residue succinyltransferase component of 2-oxoglutarate dehydrogenase complex (Dlst), mitochondrial	1,030822	2-oxoglutarate metabolic process	Chaperone binding/ Succinyltransferase activity
E9Q4D0	Proprotein convertase subtilisin/kexin type 6 (Pcsk6)	1,42278	Glycoprotein metabolic process	Heparin binding/ Endopeptidase activity
P97807	Fumarate hydratase, mitochondrial (Fh1)	1,07059	Tricarboxylic acid cycle	Fumarate hydratase activity
Q3TXS7	26S proteasome non-ATPase regulatory subunit 1 (Psmd1)	1,421197	Regulation of protein catabolic process	Enzyme regulator activity
