# Supplementary material for: Culture of Mycobacterium smegmatis in Different Carbon Sources to Induce In Vitro Cholesterol Consumption Leads to Alterations in the Host Cells after Infection: A Macrophage Proteomics Analysis
Source: Pathogens. 2021 May 28;10(6):662. doi: 10.3390/pathogens10060662 (PMC8230116; doi:10.3390/pathogens10060662)
Supplement: Supplementary file 1 [file pathogens-10-00662-s001.zip › S3_MM.pdf]

Supplementary Table S3. List of differentially regulated proteins from the group of macrophages infected with Mycobacterium smegmatis grown in minimal medium without supplementation (MM).

Uniprot acess code	Protein	*Fold-change*	Biologic Process	Molecular Function
	Up-regulated			
Q8JZZ7	Adhesion G protein-coupled receptor L2 (Adgrl2)	2,667808	Cell surface receptor signaling pathway	G protein-coupled receptor activity
E9Q9F9	Erythroid differentiation-related factor 1 (Edrf1)	1,710174	Positive regulation of transcription	Erythroid Differentiation
Q80TV8	CLIP-associating protein 1(CLASP1)	5,096183	Microtubule cytoskeleton organization	Kinetochore binding
Q9CQW9	Interferon-induced transmembrane protein (IFITM3)	1,545	Response to Interferon and receptor-mediated endocytosis	Role in the stability and function of vacuolar ATPase
